# Catechins as antibiofilm agents: molecular insights for virtual screening and in *vitro* biological evaluation

**DOI:** 10.3389/fphar.2026.1789327

**Published:** 2026-08-28

**Authors:** Adnan Amin, Ahmad Khan, Haider Ali, Wajid Zaman, Sadia Chaman, Ali Zaman, Muhammad Kashif Majeed, Niamat Ullah, Muhammad Imran Aziz

**Affiliations:** 1 Department of Life Sciences, Yeungnam University, Gyeongsan, Republic of Korea; 2 Faculty of Pharmacy, Dera IsmailKhan, Khyber Pakhtunkhwa, Pakistan; 3 Institute of Pharmaceutical Sciences, UVAS, Lahore, Pakistan; 4 Institute of Microbiology, FVAS, Gomal University, Dera Ismail Khan, Khyber Pakhtunkhwa, Pakistan; 5 Department of Chemistry, School of Natural Sciences, National University of Sciences and Technology, Islamabad (NUST), Pakistan

**Keywords:** biofilm, catechins, DFT, molecular mechanisms, quorum sensing

## Abstract

**Introduction:**

Bacterial Biofilms are of great concern because they are the main drivers of antimicrobial resistance. We investigated the potential of catechins against bacterial biofilms using advanced computational and *in vitro* models.

**Methods:**

The HPLC profiling of *Camellia sinensis* extract confirmed the presence of catechins. The ADMET and drug-likeness studies were specified to comply with the optimal values.

**Results:**

Molecular docking on 2UV0 indicated a moderate interaction with targets, which was further confirmed by DFT analysis (Homo-Lumo). Root mean square fluctuations and normal mode analysis (NMA) further supported the chemical reactivity and possible protein–ligand stabilization profile of epicatechin gallate. Antimicrobial assay showed the highest inhibition by epigallocatechin against *Pseudomonas aeruginosa* (minimum inhibitory concentration 15.6 μg/mL), followed by *Klebsiella pneumoniae* (MIC 31.2 μg/ mL), *Escherichia coli* and *Staphylococcus aureus* (MIC 62.5 μg/mL). During *in vitro* assays, epicatechin gallate showed significant inhibition of bacterial growth (for up to 20 h) and biofilm formation (61.5% ± 2.3%), while the other compounds showed slight or negligible inhibition. The ability of catechins to reduce oxidative stress and its main components in *C. sinensis* was observed.

**Conclusion:**

It was thus concluded that catechin possesses significant antibacterial and antibiofilm potential that be phenotypic response.

## Introduction

The “bacterial biofilms” pose a considerable health challenge in the modern healthcare system since these are considered the primary reason for antimicrobial resistance (Almatroudi, 2025). An overall “resistant nature” to conventional antibiotics complicates the treatment options for infections [2], thereby limiting treatment options. The biofilm formation is a multifaceted progression that includes uncontrolled microbial aggregation and the production of an extracellular matrix. This finally protects bacteria from immune responses and therapeutic agents (Flemming et al., 2023). To date, several processes involved in the development of bacterial biofilms have been described, including quorum sensing (QS), surface sensing, and the production of extracellular matrix (EPS) and intercellular second messengers (Sauer et al., 2022). Initial adhesion of bacterial cells on the target surface is “first step” in biofilm formation trailed by the production of an extracellular matrix (polysaccharides, proteins, nucleic acids) (Pugazhendhi et al., 2022). This matrix generates a shielded environment for the bacteria thereby, making them further resistant to antimicrobial agents and immune responses (Lahiri et al., 2022). Furthermore, biofilms also show reformed gene expression contours, like an upregulation of QS signaling pathways, etc (Juszczuk-Kubiak, 2024). These pathways permit bacteria within the biofilm to communicate, regulate gene expression and coordinate behaviors like production of “virulence factors” and “maturation” of biofilms (Huang et al., 2025). Aiming at both the structural integrity of the biofilm and the QS signaling mechanisms signifies a dual tactic to contest biofilm-related infections.

*Camellia sinensis* (green tea, family Theaceae) has been widely studied for its potential antimicrobial properties (Zhao et al., 2022). The leaves are rich in diverse catechins, which are polyphenolic compounds best recognized as potential anti-inflammatory, antioxidant, and antimicrobial (Teixeira and Sousa, 2021; Ayele et al., 2025). The most abundant catechins in *C. sinensis* include a diverse range, including epicatechin gallate (ECG); epigallocatechin (EGC); epicatechin (EC) and epigallocatechin gallate (EGCG) (Sangthong et al., 2024). These compounds possess a benzopyran ring structure, which is crucial for their biological activity (Wong et al., 2022). The presence of OH groups at specific positions on aromatic rings enhances their ability to scavenge free radicals, inhibiting bacterial growth and disrupting biofilm formation (Das et al., 2024; Yao et al., 2025).

Among the catechins, epigallocatechin gallate (EGCG) has fascinated researchers owing to its significant antibacterial and antibiofilm activities (Siriphap et al., 2022). EGCG has been shown to inhibit biofilm formation by various bacterial species, including *Pseudomonas aeruginosa*, *Klebsiella pneumoniae*, *Staph aureus*, and several others [17,18]. It is believed to interact with bacterial cell membranes, disrupting their integrity and hindering the production of biofilm matrix components (Pei et al., 2022; Siriphap et al., 2022; Lin et al., 2024). Moreover, EGCG has been shown to interfere with QS signaling by modulating the expression of key QS-regulated genes (Van et al., 2023). However, the molecular mechanisms underlying these effects remain only partially understood, necessitating further detailed investigation using advanced techniques, including computer simulations.

Advanced computational tools, including density functional theory (DFT), CABS-flex simulations, alongside molecular docking, do propose valuable insights into the mechanisms of action of bioactive compounds (Adedirin et al., 2025). These approaches can identify potential molecular targets and elucidate the nature of interactions and the stability of compounds with bacterial biofilms or QS signaling pathways. While some studies have examined the effects of catechins on bacterial biofilms, a comprehensive exploration of their mechanisms using computational and *in vitro* models remains limited. The research gap lies in the lack of a detailed consideration of the mechanisms of interaction between catechins and bacterial biofilm-forming systems. We hypothesize that catechins may inhibit bacterial biofilm formation through multiple mechanisms, including interference with quorum-sensing-associated pathways and redox-related bacterial processes, although the precise mode of action remains to be established. Therefore, the main objective of this study is to explore the antibiofilm potential of catechins through molecular docking, CABS-flex simulations, DFT analysis, and *in vitro* biological models.

## Materials and methods

### Sampling and extraction

Plant material was obtained from the local herbal market in D.I. Khan and authenticated and specimen authentication was performed from Islamabad Herbarium, Quaid I Azam University, Pakistan (Accession ID ISL 456127). The obtained plant material was minced to powder form and 250 g of this was macerated in 90% methanol (1L, 10 days). Finally, the extract was filtered, and plant material was re-soaked with solvent for another 10 days. The filtrate obtained from all extractions (3 times) was combined and dried using a rotary evaporator with a total yield of 35.2 g. The dried plant material was stored at 4 °C.

### HPLC analysis

An Agilent UHPLC system (1,290 Infinity II) was used during investigation that was associated with using C-18 reversed-phase column (150 mm × 4.6 mm, 5 μm; Supelco, Sigma). The mobile phase gradient contained acetonitrile (A) and acidified (0.2% acetic acid) water (B) were used as mobile phases. Filtration of the mobile phase was accomplished with a 0.2 μm nylon filter (Vertical, Bangkok, Thailand). After dissolving 10 mg/mL of the ethanolic extract of *C. sinensis*, it was further filtered using a syringe filter (0.2 μm PTFE). The sample analysis was performed at a flow rate of 1 mL/min using a DAD (diode array detector). Under the same conditions, the reference standards were analyzed, and the results were recorded.

### *In silico* analysis

#### PASS, drug likeness and ADMET analysis

Drug likeness, bioavailability, and ADMET analysis were performed using several computational tools, including pkCSM, SWISSADME, and ADMET Lab 3.0 (Naz Qaisrani et al., 2025b).

### Molecular docking

Molecular ding studies were performed to assess the interaction of catechins with target 2UV0(*P. aeruginosa* LasR/AHL complex) (Liu et al., 2012). Molecular docking was performed using AutoDock Vina (1.1.2) [23] in blind docking mode. Because the docking was conducted in blind mode, no focused redocking of the co-crystallized LasR autoinducer was performed. Therefore, the docking protocol was used only to predict possible catechin–2UV0 interactions and was not designed to validate competitive binding within the native LasR autoinducer pocket. The three-dimensional structures of all compounds were retrieved from PubChem, whereas the target protein 2UV0 was downloaded from the Protein Data Bank (PDB). The standard procedure reported earlier [23] was used to prepare the ligand and target, dock the complex, and visualize it. All generated poses were assessed for their free energies (ΔG kcal/mol) and RMSD readings. The Discovery Studio visualizer, Pymol, and Ligplot+ (Naz Qaisrani et al., 2025a) were then used to analyze each pose further and perform interaction analysis. All generated poses were appraised for their negative energies and RMSD readings by using following;

$$RMSD=\sqrt{\frac{1}{N}}\sum_{i=1}^{N}{\left(x_{i}-x_{i,ref}\right)}^{2}+{\left(y_{i}-y_{i,ref}\right)}^{2}+{\left(z_{i}-z_{i,ref}\right)}^{2}$$

Where;

RMSD values **≤** 2.0 Å specified a stable binding pose.

Xi, Yi, Zi = Coordinates of atom i in the docked pose,

N = Number of atoms in the ligand,

xi, yi, zi, ref = Coordinates of atom iii in the reference pose (e.g., lowest-energy conformation or crystallographic pose).

### Density functional theory (DFT) calculations

DFT analysis was employed using the GAUSSIAN 09 package. All theoretical calculations of compounds were determined based on B3LYP-type theory level and 6–31G (d, p) source set. The stability and reactivity of the tested compounds were determined using quantum-molecular descriptors. The resistance against disparity in the molecular electronic structure was defined by using hardness (*η*) as below (El-Shamy et al., 2022) (Equations 1–3).

$$\eta =\left(I\;-\;A\right)/2$$ (1)

Where I = −E_HOMO_ and A = −E_LUMO_, they are referred to as the energy of the maximum occupied molecular orbital, the energy of the lowest unoccupied molecular orbital, the ionization potential, and the electronic affinity, respectively. Further, the electrophilicity index (*ω*) was explained as follows.

$$w={\left(I+A\right)}^{2}/8n$$ (2)

Whereas the chemical potential (*µ*) was calculated as below;

$$µ=-\left(I+A\right)/2$$ (3)

The quantum theory of atoms in molecules (QTAIM) was used to understand better molecular interactions (Majeed et al., 2026).

## NMA simulations

The NMA (normal mode analysis) and Root mean square fluctuations (RMSF) were used to determine the flexible regions of the protein within the receptor–ligand docked complex by using CABS-fex V2.0 and the iMOD server (iMODS). Normal mode analysis and RMSF-based flexibility assessment were performed using default server parameters, and the outputs were interpreted only as predicted structural-flexibility profiles. The number of scans was unified to 10 ns? The RMSF values (Root-mean-square fluctuations) were determined from data generated by CABS-fex V2.0 and iMODs (Yamaguchi et al., 2025).

### Biological evaluation

The test samples included *C. sinensis* extract and commercial compounds, including Catechin, epicatechin gallate, epicatechin, epicatechin and epigallocatechin (Sigma Aldrich, United Kingdom).

### Antioxidant assay

Oxidative stress inhibition was assessed using the following assays.

### DPPH free radical scavenging assay

The DPPH assay was performed using an adapted method (Hussen and Endalew, 2023). Briefly, a fresh DPPH solution in methanol (50 μL; 0.1 mM) was arranged in a 96-well microplate and mixed with the sample (50 μL; 100–1.56 μg/mL). The mixture was incubated in a dark cabinet for 30 min. Afterwards, reading (absorbance) was measured at 517 nm using a microplate reader. The control was prepared following the same procedure, with Trolox as the standard. Percentage (%) inhibition was calculated as follows:

$$\%\text{ inhibition}\left(1-\frac{A_{a}}{A_{b}}\right)\times 100$$

Where *Aa* represents the sample absorbance, and Ab indicates the control absorbance. IC_50_ values for the tested samples were calculated from linear plots.

### FRAP assay

The FRAP (Ferrous-reducing antioxidant assay) was performed by using an established procedure with slight modifications (Youn et al., 2019). In brief, the FRAP reagent was prepared using FeCl3.6H_2_O (10 mL, 20 mM), TPTZ solution (10 mM, 10 mL) and acetate buffer (100 mL, 300 mM). The FRAP reagent (2,850 µL) was then mixed with test samples (150 μL; 25 µg) and placed untouched in the dark for at least 45 min. Finally, the absorbance was recorded at 593 nm. A Trolox calibration curve (0–250 μg/mL) was generated to finalize the results (mg TAE/Gram sample).

### Phosphomolybdenum assay

The phosphomolybdenum assay was performed by a modified method (Diwan et al., 2012). In brief, an aliquot part of test sample (0.1mL; in MeOH) was placed carefully in a vial (3 mL capacity) already having 1 mL of reagent (0.6 M H_2_SO_4_, 28 mM NaHPO_4_.7H_2_O, and 4 mM (NH_4_)2 MoO_4_, trailed by incubation at 95 °C for 90min (water bath). Afterwards, test samples were shaken slightly and permitted to cool at room temperature. Finally, the absorbance of the mixture was noted at 765 nm using UV spectrophotometer. The following formula was designated to calculate the percent inhibition.

$$\%\text{ inhibition}\left(1-\frac{A_{a}}{A_{b}}\right)\times 100$$

Where *Aa* represents the sample absorbance, and Ab represents the control absorbance.

### Hydrogen peroxide scavenging activity

The H_2_O_2_ assay was performed by standard procedure (Bhatti et al., 2015). Briefly, the test sample (EOs) or standard (ascorbic acid) solution (400 μL at various concentrations) were reacted with H_2_O_2_ solution (600 μL, 2 mM). The mixture was moderately vortexed for 1 min and left undisturbed for 15 min. Absorbance was recorded at 230 nm using a UV spectrophotometer. Finally, the percentage % of inhibition was designed as;

$$\%\text{ inhibition}\left(1-\frac{A_{a}}{A_{b}}\right)\times 100$$

Where *Aa* represents the sample absorbance, and Ab represents the control absorbance.

### Antimicrobial evaluation

#### Determination of MIC (minimum inhibitory concentrations)

A modified methodology was used to evaluate the antibacterial properties of compounds (Elshikh et al., 2016). In the MIC assay, 50 μL of a 24-hour-grown bacterial strain was added to each of the 96 microwell plates, followed by 50 μL of the test sample. For 24 h, the plates were incubated at 37 °C. The next day, each well received 40 μL of resazurin solution, which was incubated for 60 min at 37 °C. A microplate reader containing 96 plates was used to record colorimetric readings (Hippo MPP-96, Biosan).

#### Antibiofilm activities

A modified methodology was adopted for the biofilm evolution experiment, using 12-well polystyrene plates (Richter et al., 2023). The bacterial strain (10^6^ CFU/mL) was inoculated with 100 μL of the test sample followed by incubation for 24–48 h by inoculating it into medium (280 μL) at an initial turbidity of 0.5 at 600 nm. Plates’ cell growth was assessed at 592 nm. Crystal violet was used to measure biofilms in 12-well plates. The biofilm was then measured by absorbance at 592 nm after 95% ethanol was applied to the identified cell. Lastly, the percentage % of inhibition was designed as;

$$\%\text{ inhibition}\left(1-\frac{A_{a}}{A_{b}}\right)\times 100$$

Where *Aa* represents the sample absorbance, and Ab represents the control absorbance.

#### Growth curve

Time-kill kinetic studies were performed using the standard method (Qureshi et al., 2022). Briefly, test samples were cultured with *P. aeruginosa* (10^6^ CFU/mL) and incubated at 37 °C. Afterwards, the samples were collected at different time points (0–24 h), then loaded onto Mueller-Hinton agar plates and incubated at 30 °C for 24 h. A control sample (without a test sample) was taken and processed in parallel. The results were graphed as log CFU/mL versus time (0–24 h).

#### Statistical analysis

The means of at least three experiments, along with the standard deviation, is used to express all experimental results. A statistically significant difference was p < 0.05, and SPSS software version 24.0 was employed to conduct statistical data analysis. Where applicable, the Student’s t-test and the analysis of variance (ANOVA)/*post hoc* analysis using Tukey’s HSD test were used.

## Results and discussions

### HPLC analysis and standardization

**FIGURE 1:**
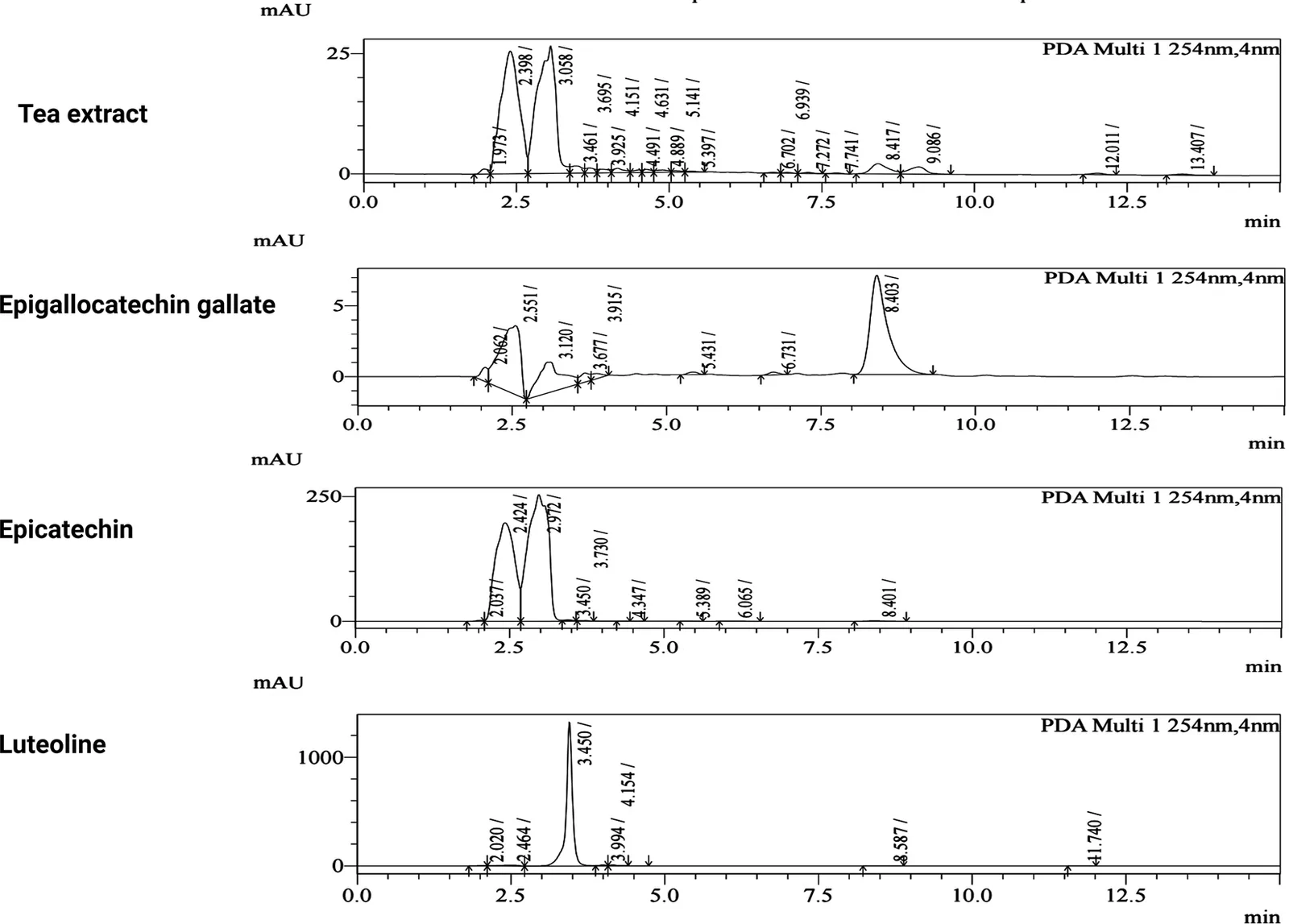
HPLC analysis of Tea extract and major compounds.

**FIGURE 2:**
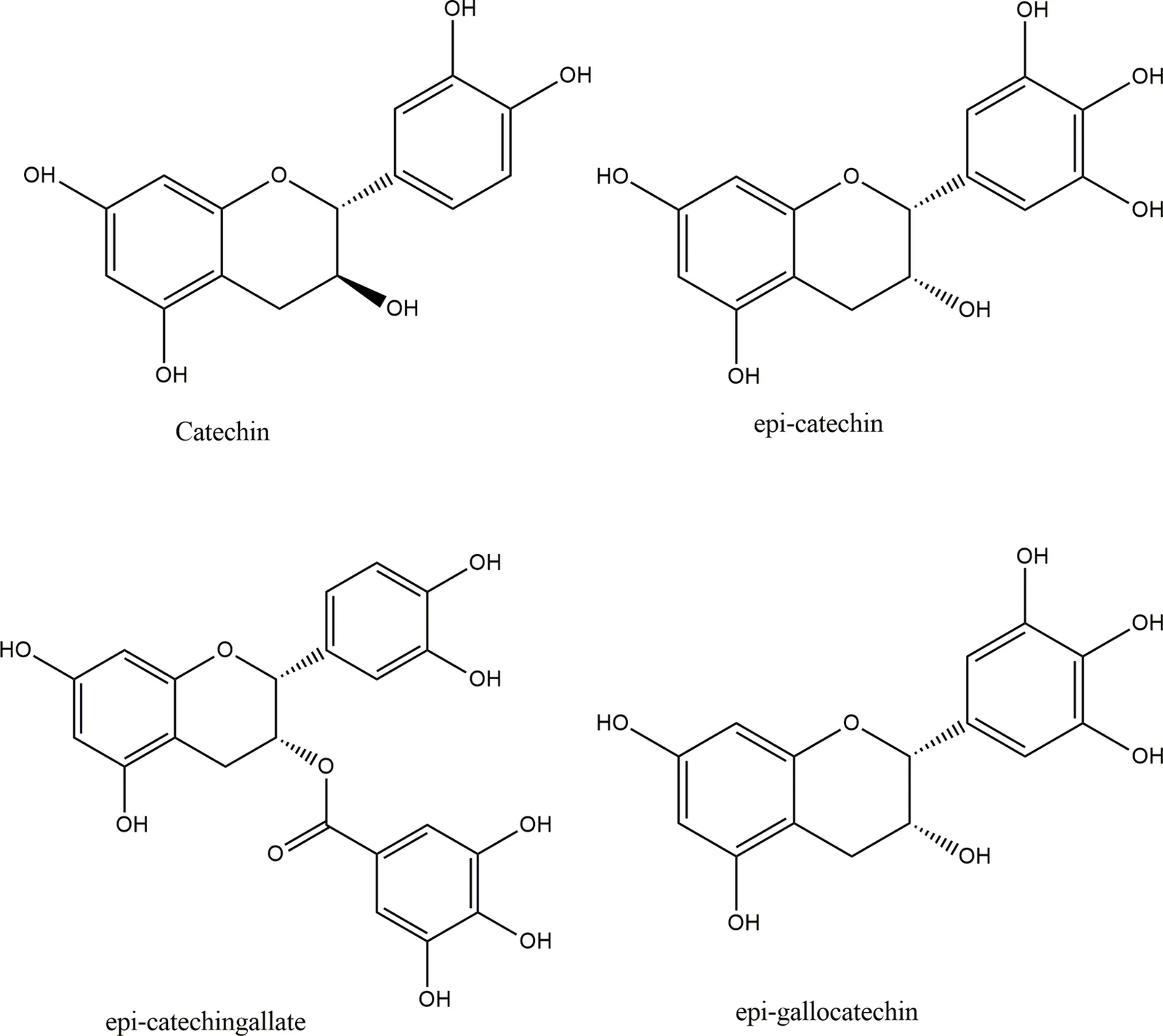
Structure of analyzed compounds.

HPLC profiling of *Camellia sinensis* extract was performed using a PerkinElmer HPLC. The standard curve (calibration curve) was constructed from stock solutions readings using serial dilution of aliquots. The retention time of epicatechin gallate was 8.4 min, as shown in the crude extract chromatograms (Figures 1, 2). Based on the standard calibration curve, *Camelia sinensis* extract was standardized to contain epicatechin gallate (35.2 mg/g); epicatechin (3.4 mg/g), catechin (1.3 mg/g) epigallocatechin (11.2 mg/g) and luteolin (0.82 mg/g).

### Computational analysis

#### Drug likeness properties, ADMET, and molecular docking

**FIGURE 3:**
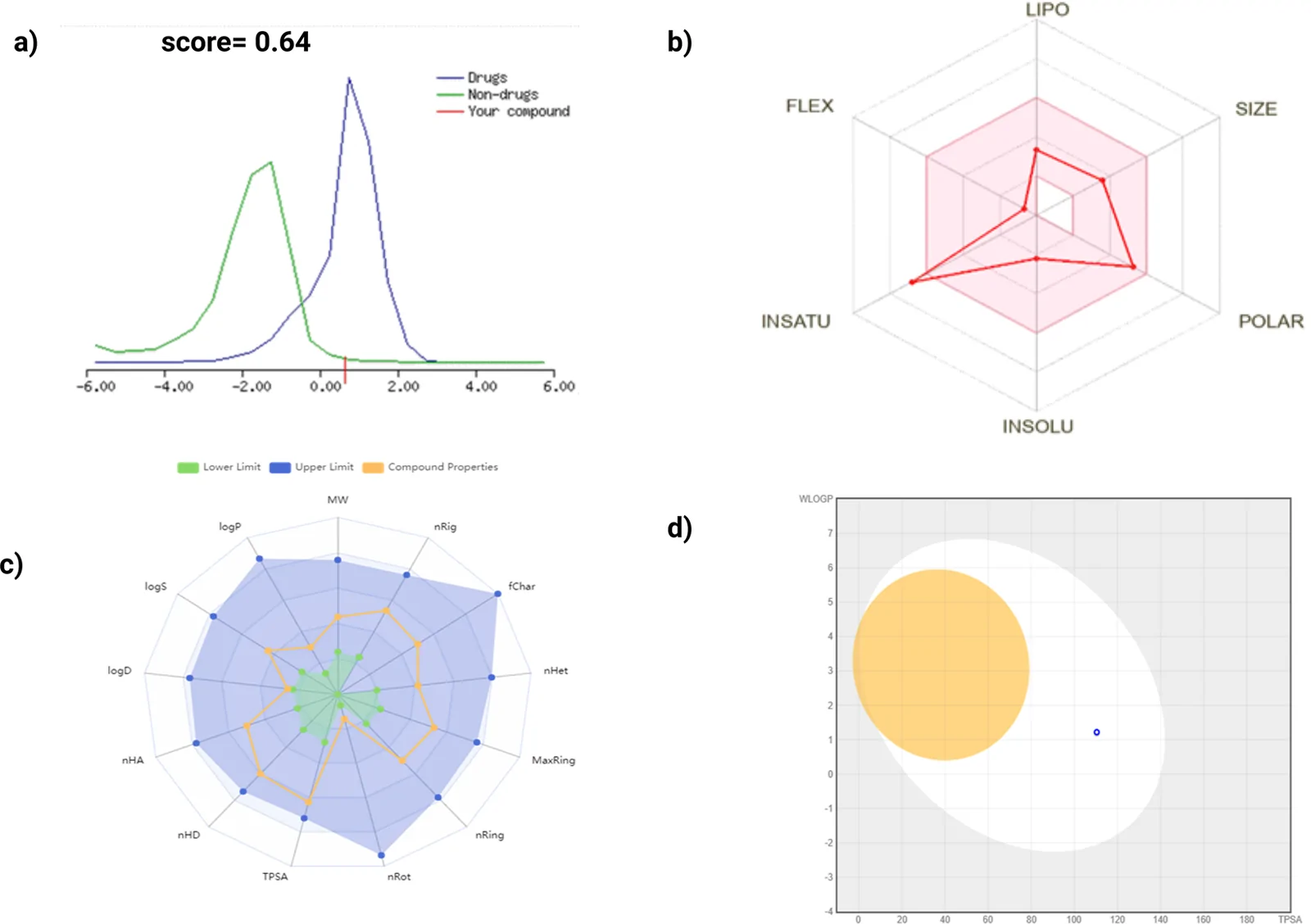
Drug likeness scores **(a)**, bioavailability radar **(b)**, ADMET analysis **(c)**, and Boiled egg model **(d)** for catechin.

**FIGURE 4:**
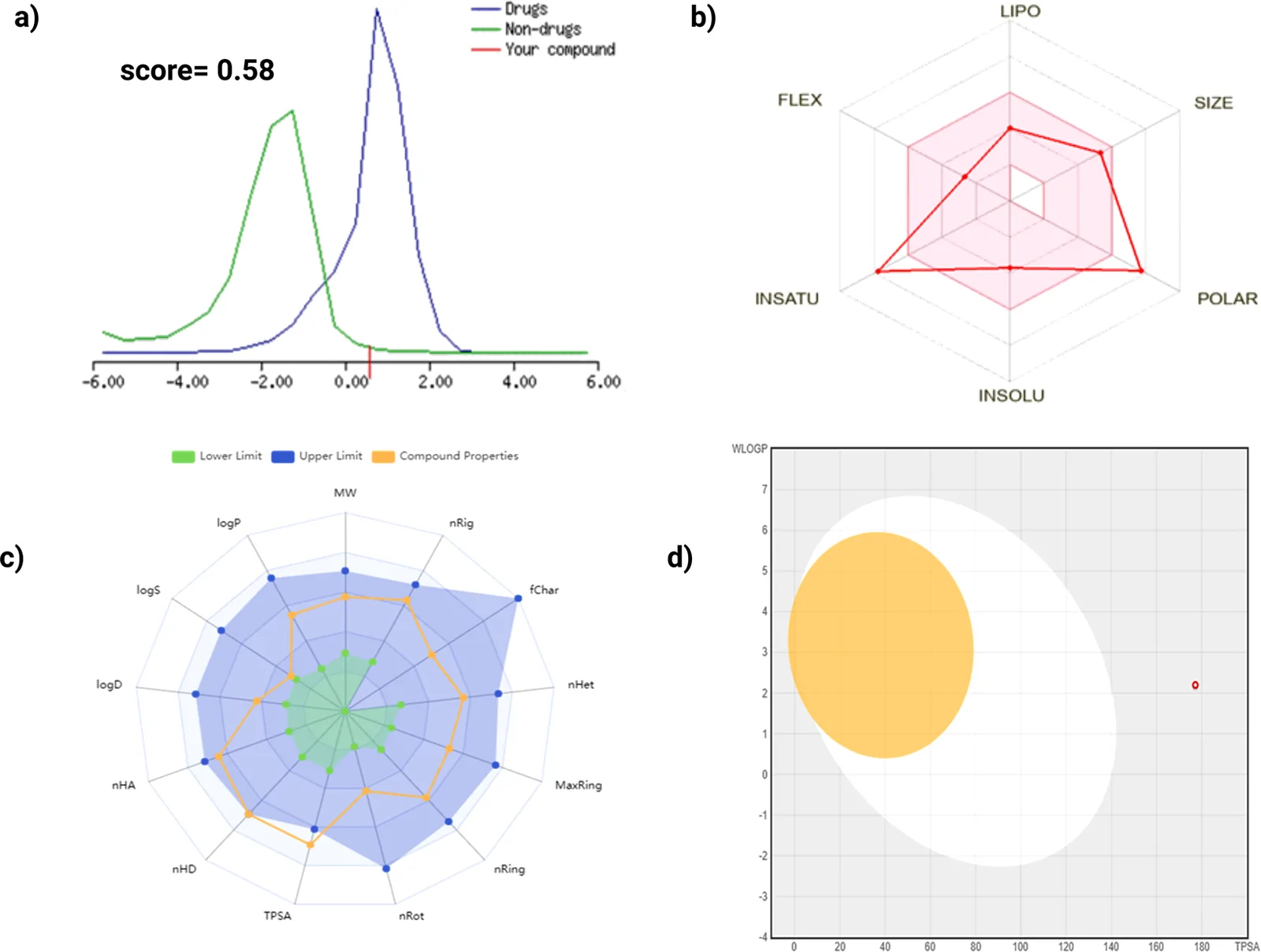
Drug likeness scores **(a)**, bioavailability radar **(b)**, ADMET analysis **(c)**, and Boiled egg model **(d)** for epicatechingallate.

**FIGURE 5:**
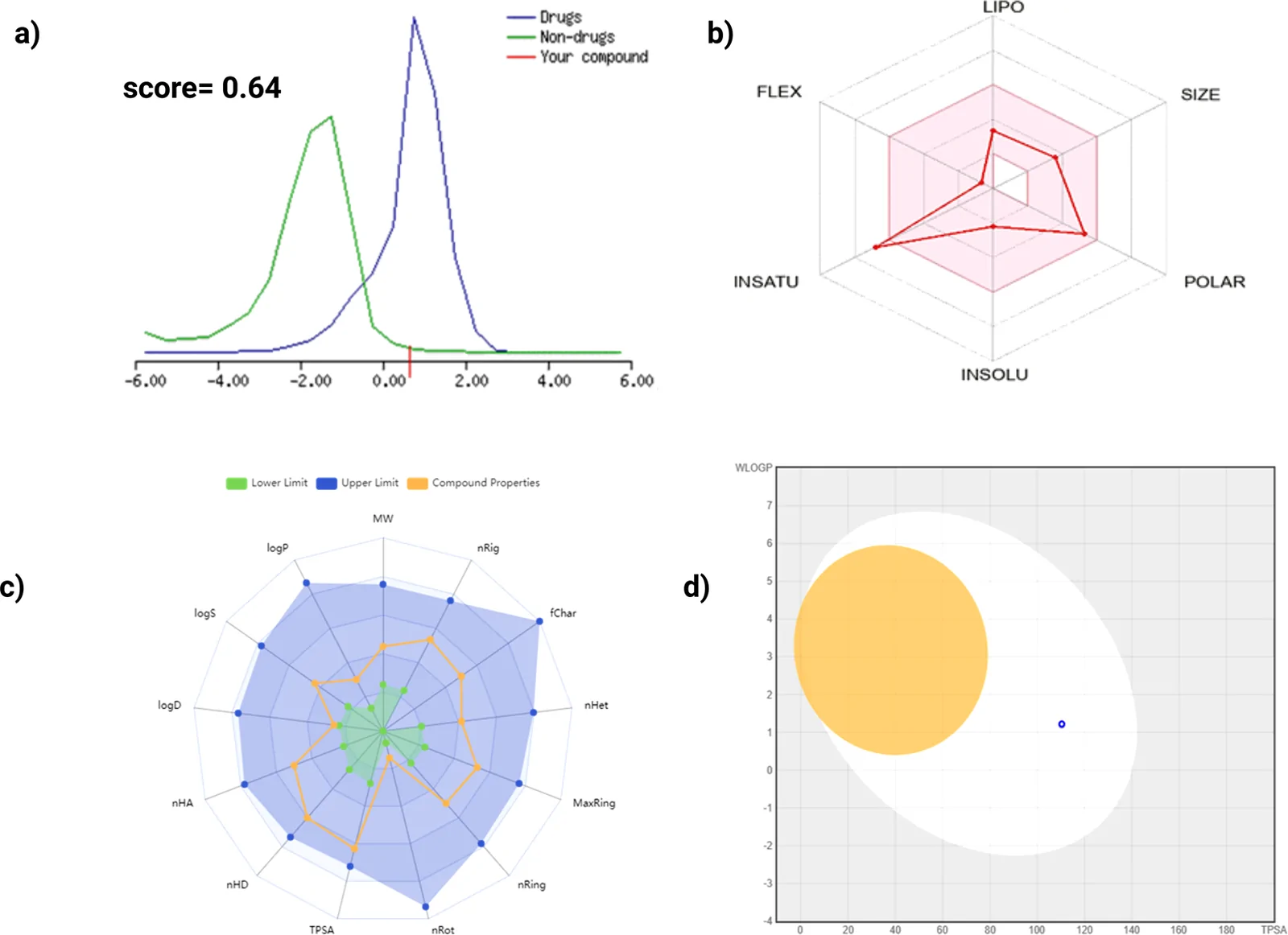
Drug likeness scores **(a)**, bioavailability radar **(b)**, ADMET analysis **(c)**, and Boiled egg model **(d)** for epicatechin.

**FIGURE 6:**
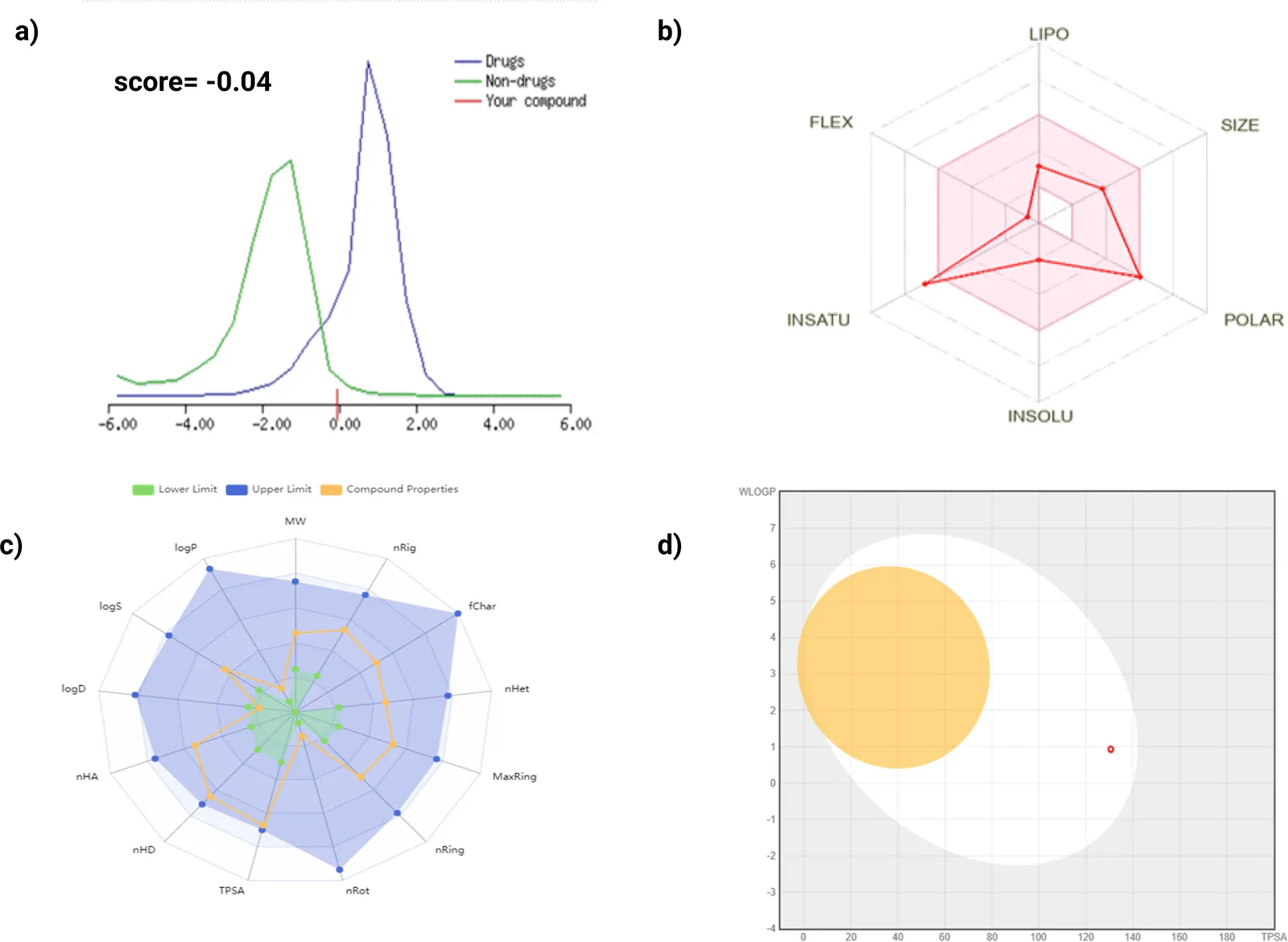
Drug likeness scores **(a)**, bioavailability radar **(b)**, ADMET analysis **(c)** and Boiled egg model **(d)** for epigallocatechin.

*In silico* analysis of compounds was performed using the computational tools described earlier. The PubChem was used to obtain structures and SMILES of the compounds (Table 1). The drug-likeness properties of molecules were examined using Lipinski’s rule of five, and drug-likeness scores were recorded (Table 2). The drug-likeness properties of the analysed compounds revealed that epicatechin gallate and epigallocatechin showed one violation, i.e., the number of H-bond donors (<5). Moreover, the drug-likeness scores of all analyzed compounds were within the allowed range (±1), indicating that all tested molecules meet the criteria for drug-likeness (Figures 3–6).

**TABLE 1:** SMILES of analyzed catechins.

Sample	SMILES
Catechin	C1C(C(OC2 = CC(=CC(=C21)O)O)C3 = CC(=C(C=C3)O)O)O
Epicatechin gallate	C1C(C(OC2 = CC(=CC(=C21)O)O)C3 = CC(=C(C=C3)O)O)OC(=O) = CC(=C(C(=C4)O)O)O
Epicatechin	C1C(C(OC2 = CC(=CC(=C21)O)O)C3 = CC(=C(C=C3)O)O)O
Epigallocatechin	C1C(C(OC2 = CC(=CC(=C21)O)O)C3 = CC(=C(C(=C3)O)O)O)O

**TABLE 2:** Drug likeness properties of catechins, gallocatchins.

Sample	Score	M.Wt	M.formula	Log p	H acceptor	H donor	Molar refractivity (cm^3^)	Violations
Catechin	0.64	290.08	C_15_ H_14_ O_6_	0.53	6	5	73.3	0
Epicatechingallate	0.58	430.09	C_21_ H_18_ O_10_	0.86	10	7	106.9	1
Epicatechin	0.64	290.08	C_15_ H_14_ O_6_	0.53	6	5	73.6	0
Epigallocatechin	−0.04	306.07	C_15_ H_14_ O_7_	0.26	7	6	108.4	1

The ADMET (absorption, distribution, metabolism, and elimination) was performed by using SWISS ADME and ADMET Lab 3.0 tools. All tested molecules had a high polar surface area (TpSA), indicating their polarity. Further, none of the molecules were able to cross the blood-brain barrier and thus were not expected to affect the CNS. All other physicochemical parameters were within the permissible range (Table 3; Figures 3–6). ADMET profiling further indicated that epigallocatechin may be absorbed via the oral route, whereas all other molecules exhibited limited oral absorption. Catechin and epicatechin acted as Pgp substrates, and none of the tested molecules were either substrates or inhibitors of the Cytochrome p450 family, which shows little or no metabolism in the liver. In addition, low skin permeation was observed for all tested compounds, which were non-toxic in the Ames test and non-hepatotoxic in hepatotoxicity predictions (Table 4).

**TABLE 3:** Bioavailability properties of catechins, gallocatchins.

Sample	MolPSA (A^2^)	Mol Vol (A^3^)	pKa<0	BBB	Steriocenters
Catechin	90.45	261.13	9.52	2.73	2
Epicatechingallate	140.41	400.87	8.07	1.70	2
Epicatechin	90.45	261.13	9.52	2.73	2
Epigallocatechin	105.93	271.81	9.36	2.50	2

**TABLE 4:** ADMET profiling of catechins, gallocatchins.

Sample	Catechin	Epicatechingallate	Eipcatechin	Epigallocatechin
GI absorbtion	Low	Low	Low	High
BBB permeant	No	No	No	No
Pgp substrate	Yes	No	Yes	No
CYP1A2 inhibitor	No	No	No	No
CYP2C19 inhibitor	No	No	No	No
CYP2C9 inhibitor	No	No	No	No
CYP2D6 inhibitor	No	No	No	No
CYP3A4 inhibitor	No	No	No	No
Log Kp (skin permeation)	−7.82	−7.91	−7.82	−8.17
AMES toxicity	0.52(No)	0.56(No)	0.525(No)	0.577(No)
Hepatotoxicity	0.5 (no)	0.203(No)	0.5 (no)	0.436(No)

As obvious from our findings that ADMET analysis is favorable for oral absorption for epicatechin gallate and other catechins. However, it is noteworthy these compounds have low skin permeation in the analysis, thus making them more suitable for topical applications. The bioavailability properties of catechins do suggest their role as adjunctive agents for localized therapy, particularly for chronic infections and biofilm-related conditions where topical delivery would be more beneficial. A low skin permeation observed for the catechins supports their use in such as gels or creams, for localized treatment of chronic infections associated with biofilms.

#### ProTox-II toxicity analysis

**FIGURE 7:**
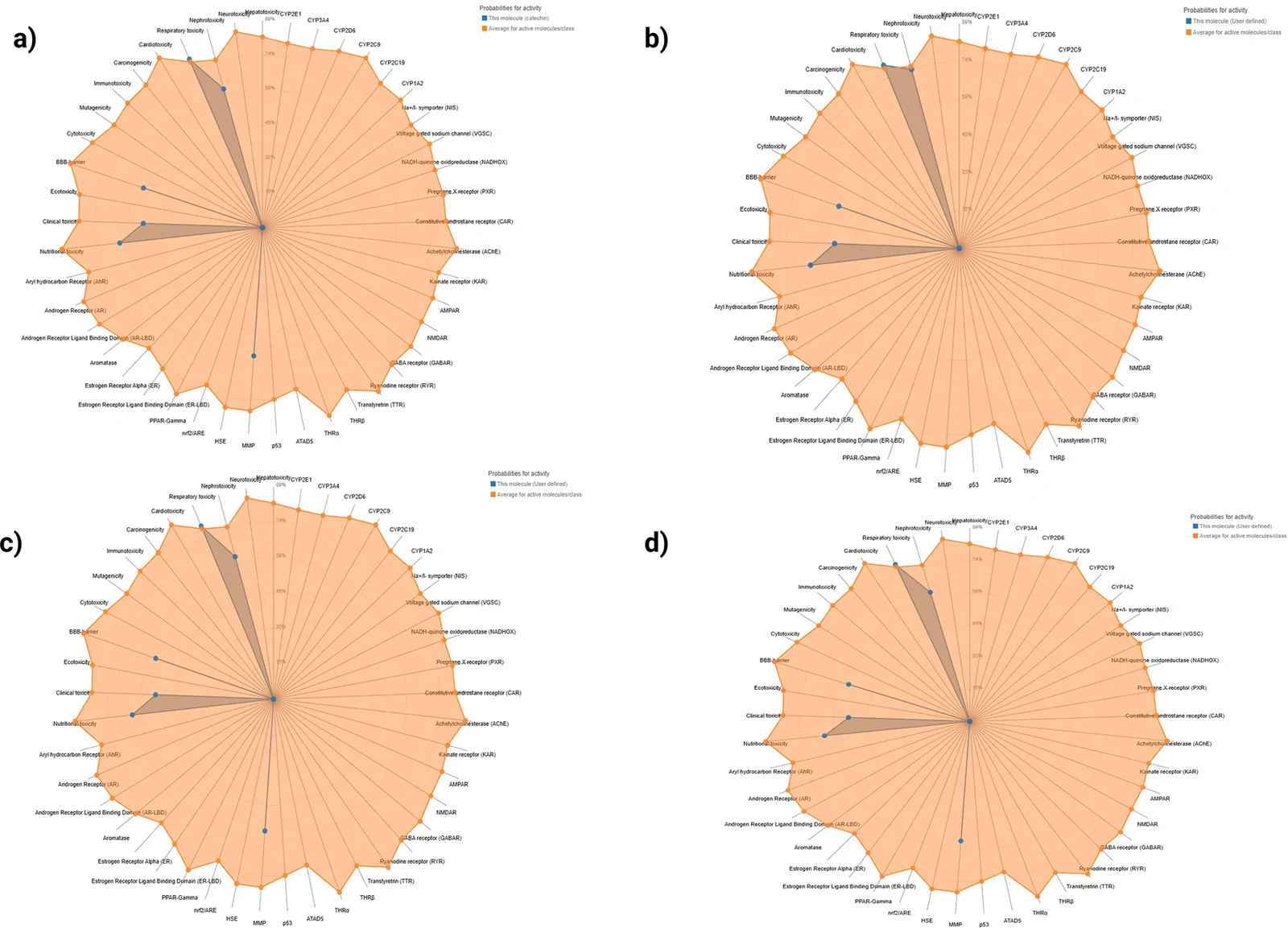
ProTox-II Toxicity analysis of all analyzed compounds, including catechin **(a)**, epicatechin gallate **(b)**, epicatechin **(c)**, and epigallocatechin **(d)**.

The Protox-II online server was used for drug analysis and to assess cytotoxicity, mutagenicity, and hepatotoxicity. This method is central to drug design, as it provides a quicker and more robust validation of compound toxicity. It was noticed that all tested chemicals and medications are nontoxic (Table 5; Figure 7).

**TABLE 5:** Toxicity prediction analyses.

Compounds	Hepatotoxicity	Mutagenicity	Cytotoxicity
Catechin	No	No	No
Epicatechin gallate	No	No	No
Eipcatechin	No	No	No
Epigallocatechin	No	No	No

#### Molecular docking

**FIGURE 8:**
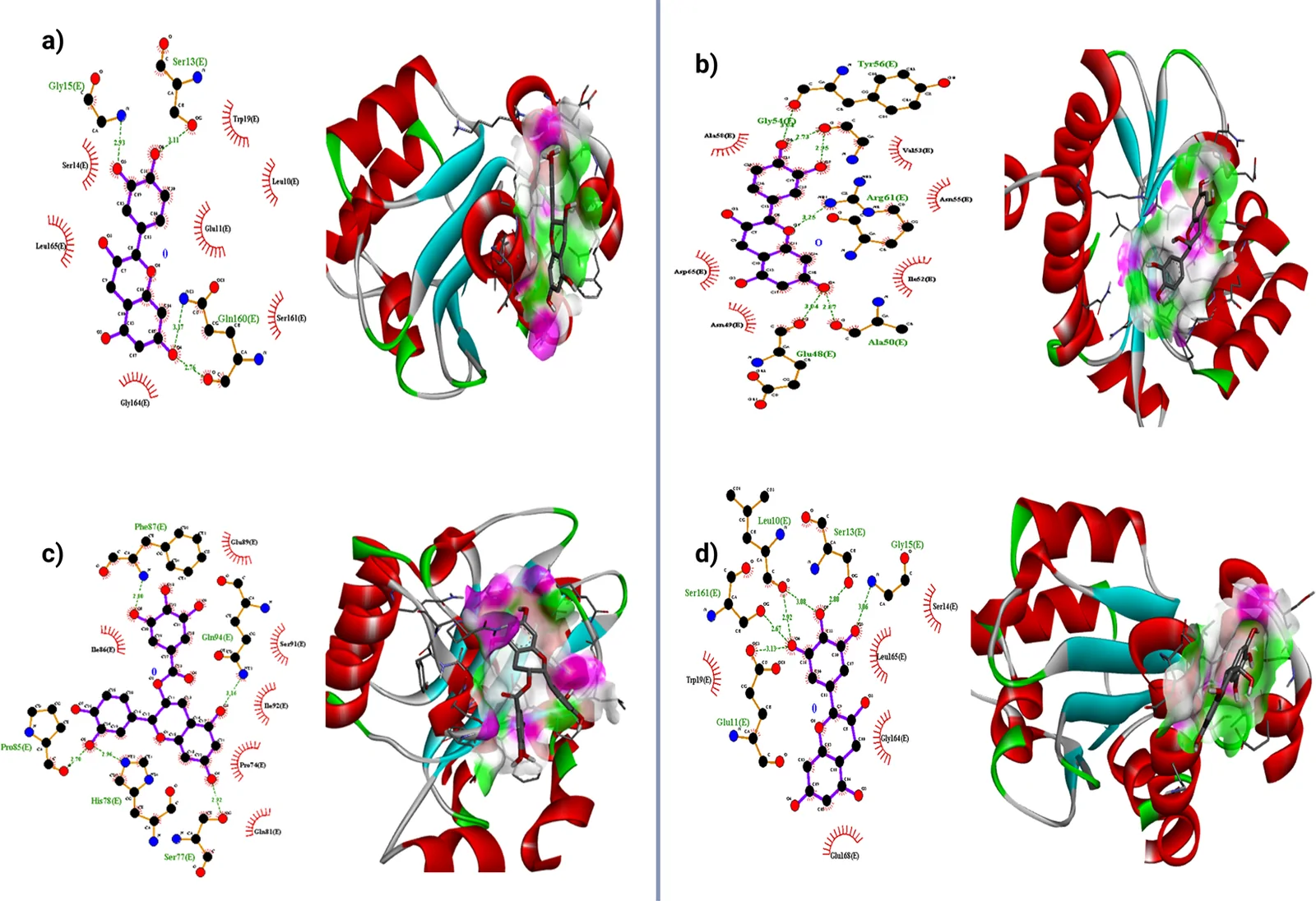
Docking of **(a)** catechin, **(b)** epicatechin **(c)** epicatechin gallate, and **(d)** epigallocatechin with 2UV0 and interaction analysis showing H-bonding and hydrophobic interactions.

Molecular docking against the *P. aeruginosa* LasR ligand-binding domain structure 2UV0 was performed for all tested compounds. This regulator was selected based on earlier investigations pertaining to biofilm and Quorum sensing (Emara et al., 2025). The docking was conducted in blind mode to identify possible catechin interaction regions on the protein, rather than to test competitive occupation of the native acyl-homoserine lactone binding pocket. Therefore, no assumption of competition with the native autoinducer was made. Epicatechin gallate showed the most favorable predicted binding energy (−7.5 kcal/mol), among the tested compounds, followed by catechin (−7.0 kcal/mol), epicatechin (−6.8 kcal/mol), and epigallocatechin (−6.0 kcal/mol) (Table 6; Figure 8). These docking results indicate predicted catechin–2UV0 interactions, but they do not establish competitive LasR inhibition or displacement of the native autoinducer.

**TABLE 6:** Docking scores and interaction analysis of tested catechins and gallates against 2UV0.

Sample	Affinity (kcal/mol)	H-bond interactions	Residual hydrophobic interactions
Catechin	−7.0	Gln160, Gly15, Ser13	Gly164, Leu165, Ser14, Trp19, Leu10, Glu11, Ser161
Epicatechin gallate	−7.5	Ser77, His78, Pro85, Phe87, Gln94	Ile86, Glu89, Ser91, Ile92, Pro74, Gln81
Epicatechin	−6.8	Glu48, Ala50, Gly54, Tyr56, Arg61	Asn49, Asp65, Ala58,Val53, Asn55, Ile52
Epi gallocatechin	−6.6	Glu11, Ser161, Leu10, Ser13, Gly15	Trp19, Ser14, Leu165, Gly164, Glu168

Among all tested compounds, epicatechin gallate showed the moderate interaction energy (−7.5 kcal/mol), due to interactions involving five amino acid residues, including Ser77, His78, Pro85, Phe87, and Gln94. This interaction was further stabilized with hydrophobic interactions (Van der Waals interactions) that appeared due to Ile86, Glu89, Ser91, Ile92, Pro74, and Gln81 (Table 6). The epigallocatechin also showed moderate interaction with target 2UV0 with high free energy interaction energy (−6.6 kcal/mol. The amino acids, including Glu11, Ser161, Leu10, Ser13, Gly15, interacted with the target protein through H-binding, whereas Trp19, Ser14, Leu165, Gly164, and Glu168 interacted within the complex through Van der Waals, π-anion, and π- π-alkyl and amide-π-stacked interactions (Table 6; Figure 8).

Molecular docking of catechin indicated H-bonding interactions with Gln160, Gly15, and Ser13 amino acids, and hydrophobic interactions with Gly164, Leu165, Ser14, Trp19, Leu10, Glu11, and Ser161 Amino acids. The hydrophobic interactions included Van der Waals, π-anion, and π- π-sigma and amide-π-stacked interactions. The interaction was supported with moderate free energy (−7.0 kcal/mol). The epimer of this compound, i.e., epicatechin, showed slightly variable results. In this case, the complex was supported by five H-binding interactions with Glu48, Ala50, Gly54, Tyr56, and Arg61, with a slightly lower free energy (−6.8 kcal/mol). The protein folding was supported by Van der Waals π-anion and π- π-alkyl interactions that were shown by Asn49, Asp65, Ala58, Val53, Asn55, and Ile52 amino acids (Table 6; Figure 8). A change in docking pattern was attributed to a change in the orientation of the OH group (epimer form) (Zhang et al., 2018). For a significant docking, stable complex formation with high specificity is very important, which is mainly driven by both hydrophobic and H-bonding interactions (Vaidyanathan et al., 2023). In this case, the ligand-protein complex is stabilized by both hydrogen Bonding and hydrophobic interactions. Thus, the docking profile supports epicatechin gallate as a candidate LasR-interacting molecule; however, the biological activity observed *in vitro* cannot be attributed specifically to competitive LasR binding without additional validation.

#### DFT calculations

**FIGURE 9:**
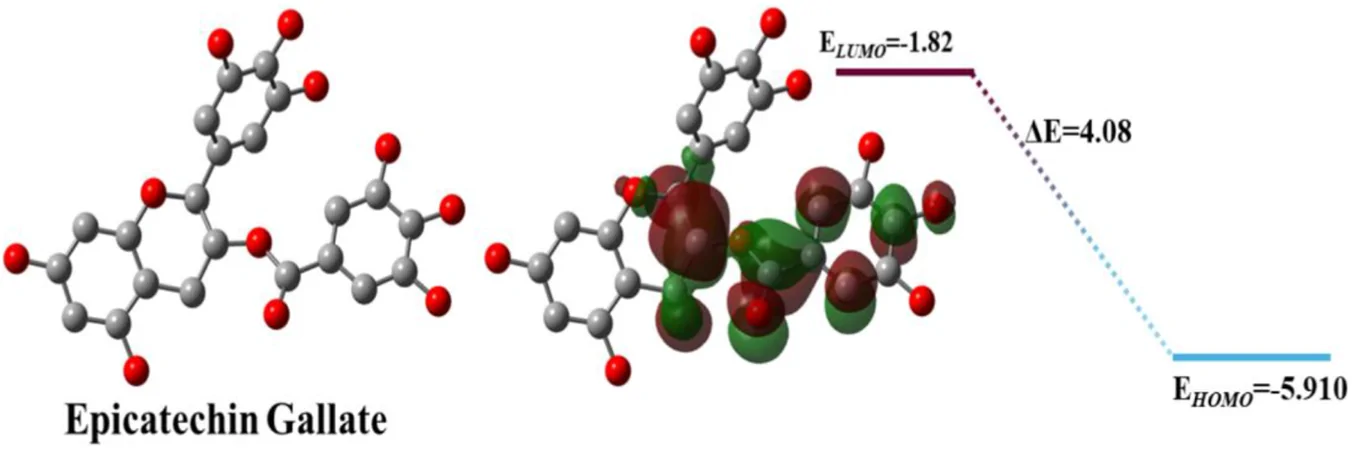
DFT calculation for epigallocatechin with HUMO and LUMO analysis.

The quantum chemical descriptors for epicatechin gallate (ECG) were calculated using Density Functional Theory (DFT) at the RB3LYP/6–31G (d, p) level. The EHOMO and ELUMO energies were determined to be −5.90 eV and −1.82 eV, respectively, resulting in a narrow HOMO–LUMO gap (E.g., = 4.08 eV). Using Koopmans’ theorem, the ionization potential (I ≈ 5.85 eV) and electron affinity (A ≈ 3.44 eV) were computed from the HOMO and LUMO energies. The chemical hardness (ƞ = 1.20 eV) and electrophilicity index (ω = 8.95 eV) further characterize the chemical behavior of ECG. Finally, the dipole moment of EGC (µ ≈ −4.64 Debye) indicates considerable polarity (Table 7; Figure 9).

**TABLE 7:** DFT calculation of compounds.

Sample	HOMO	LUMO	E (RB3LYP)	EHOMO	E LUMO	E.g.,	I	A	µ	ƞ	ω
Epi catechin-gallate	−0.21700	−0.067	−1,099.805	−5.90	−1.82	4.8	5.85	3.44	−4.64	1.20	8.95

The quantum chemical descriptors calculated for epicatechin gallate (ECG) provide valuable insights into its potential to bind LasR and inhibit biofilms. The small HOMO-LUMO gap (4.08 eV) suggests high chemical reactivity (Shahab and Husain, 2021), consistent with ECG’s ability to engage in electron transfer, a critical step in interactions with biological targets. The electrostatic potential map highlights regions of negative charge that can interact with the positively charged regions of the LasR receptor, facilitating binding (Kangas and Tidor, 1998). Furthermore, ECG’s hydrogen bonding regions, particularly around the hydroxyl groups, are capable of forming strong hydrogen bonds with donor and acceptor sites on the LasR protein, as indicated by the molecular dipole (µ = −4.64 Debye). The low chemical hardness (ƞ = 1.20 eV) and high electrophilicity index (ω = 8.95 eV) suggest that ECG is highly polarizable and reactive, which enhances its ability to form stable complexes with LasR and potentially disrupt biofilm formation (Talmaciu et al., 2016). These results link the electronic properties of ECG to its anticipated biological activity, supporting its potential as a biofilm inhibitor and LasR binding agent.

Several DFT investigations on natural flavonoids reveal analogous electronic patterns. For example, Hou et al. conducted DFT analyses across 25 tea flavonoids and showed that HOMO and LUMO orbitals are key indicators of antioxidant activity; flavonoids with higher HOMO energies and smaller gaps displayed increased reactivity and free radical scavenging potential. Likewise, electrostatic and frontier orbital analyses of related phenolic compounds demonstrate that electron-donating ability, as reflected in ionization potentials and orbital energies, is a central determinant of reactivity in biological contexts (Kamel et al., 2023). Together, these electronic descriptors are consistent with the observed docking trend, but they do not define whether ECG binds an active, allosteric, or native autoinducer pocket of LasR.

#### Normal mode analysis (NMA)

##### CABS-flex 2.0

**FIGURE 10:**
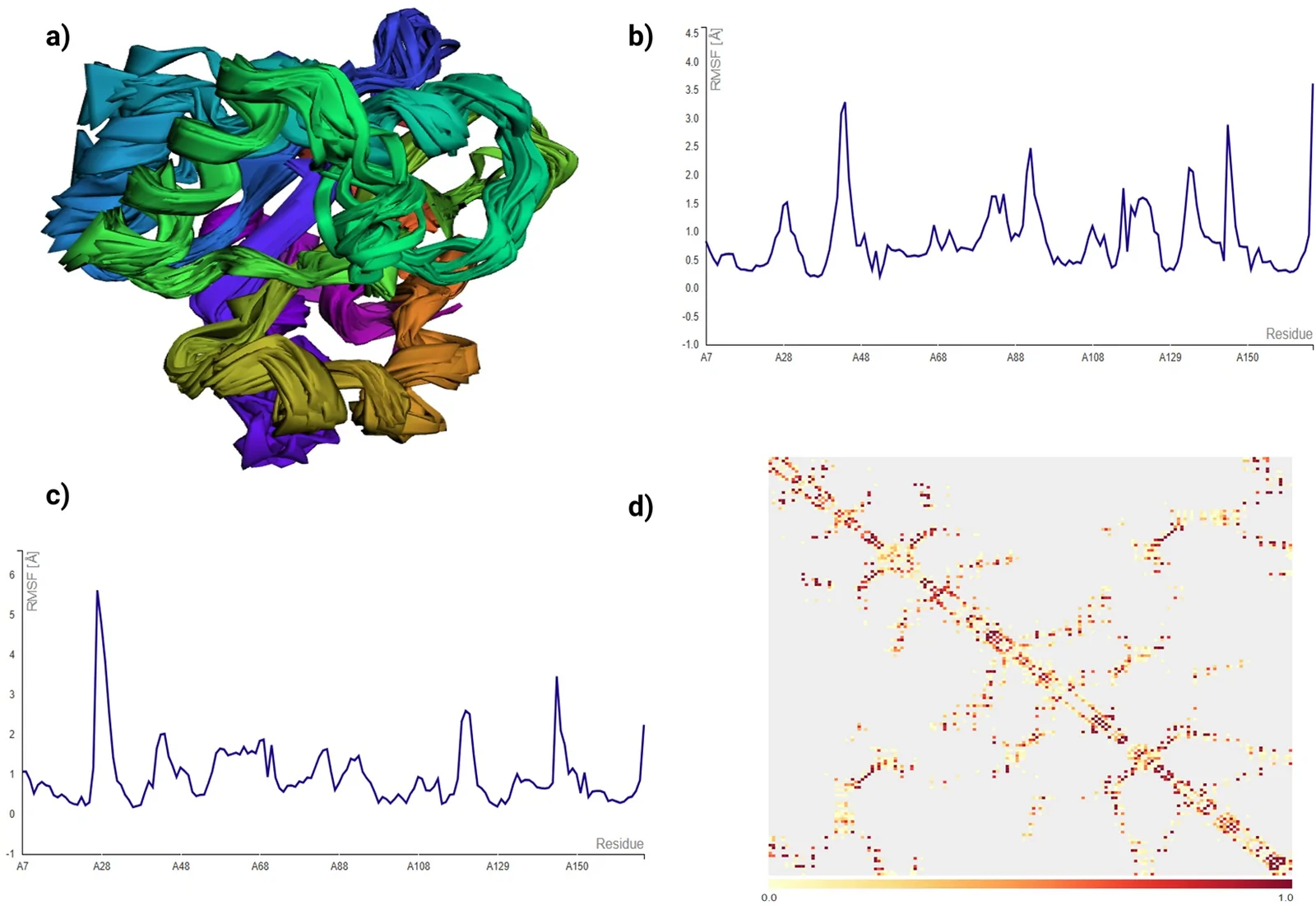
Outcomes of NMA simulations results using CABS-fex. Tool. **(a)** Multimodel superimposed simulated structure of ligand-protein complex **(b)** Fluctuation map of 2UV0 target and RMSF profile **(c)** Fluctuation map of epicatechin gallate-2UV0 complex and RMSF profile **(d)** Contact map of epicatechin gallate-2UV0 complex.

The highest variability in the RMSF of the protein (2UV0) was observed for residues 6 (3.139 Å), 7 (0.827 Å), 8 (0.655 Å), 9 (0.512 Å), and 10 (0.410 Å). On the other hand, the epicatechin gallate-2UV0 complex showed a marked decrease in RMSF values in residues 6 (2.713 Å), 7 (1.061 Å), 8 (1.065 Å), 9 (0.858 Å), and 10 (0.513 Å). Notably, residues 6, 7, and eight exhibited a significant reduction in flexibility upon binding with epicatechin gallate, indicating a stabilizing effect on the protein structure. The RMSF difference values, such as the 0.234 Å difference for residue 7 and the 0.410 Å difference for residue 8, further emphasize the stabilizing role of the epicatechin gallate binding (Figure 10).

##### iMOD server

**FIGURE 11:**
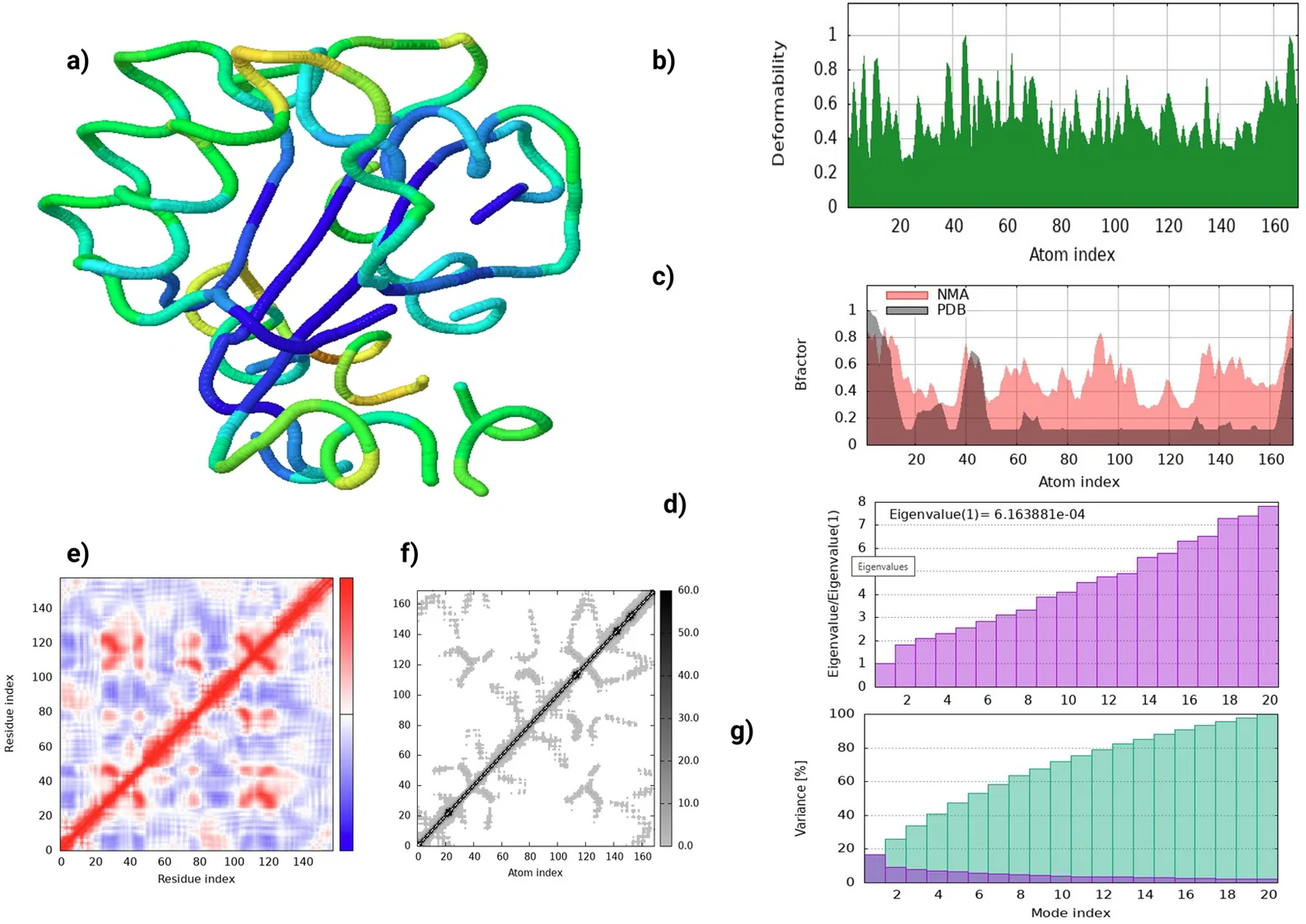
Outcomes of NMA simulations results using the iMODs tool. The Normal Mode Analysis epigallocatechin-2UV0 and various parameters, including **(a)** complex motion tolerance, **(b)** deformability, **(c)** B-factor plot, **(d)** eigenvalues, **(e)** covariance map, **(f)** elastic network model, and **(g)** variance.

Normal mode analysis using iMODS was used to evaluate the collective flexibility of the predicted 2UV0–epicatechin gallate complex. The deformability profile indicated that mobility was not uniformly distributed across the protein, suggesting that ligand binding may restrict selected flexible regions while preserving global protein motion. The B-factor/RMSF pattern supported this interpretation by showing residue-level differences in predicted fluctuation, whereas the covariance map indicated coordinated and anti-correlated residue motions within the elastic network. The eigenvalue reflected the energy required to deform the complex; therefore, a higher eigenvalue would indicate greater resistance to conformational deformation and higher predicted stiffness. However, iMODS is a coarse-grained normal mode analysis tool and should be interpreted as a structural-flexibility assessment rather than validation of docking accuracy, competitive binding, or functional LasR inhibition (López-Blanco et al., 2014; Bauer et al., 2019).(Figure 11). These normal mode analysis results suggest that the predicted protein–ligand complex did not show major global disruption in the modeled protein flexibility profile. However, because focused native-ligand redocking was not performed, these data should not be interpreted as validation of a correct docking pose or as evidence of competitive binding within the native LasR autoinducer pocket.

### *In vitro* screening

#### Antioxidant activities

The oxidative stress-lowering capacity of *C*. *sinensis* and its major components was evaluated using diverse antioxidant mechanisms, including DPPH, H_2_O_2_, Phopohomolybdenum, and FRAP assays. A significant antioxidant potential was noticed in all samples (Table 8). The oxidative stress-lowering capacity was in the following Order: epi-catechin gallate > Epi-gallocatechin > Epi-catechin > catechin. The antioxidant capacity of *C. sinensis* extract was slightly lower based on fact that individual compounds concentration in total extract is lower compared to individually analyzing compounds. In case of compounds, it has been reported that the position of OH groups and the number of hydroxyl groups in the catechins play a substantial role in antioxidant activity (Parcheta et al., 2021; Wang et al., 2021). Further antioxidant activity may be higher due to the presence of gallate functional groups on catechins (Caturla et al., 2003; Yong et al., 2022). Similarly, the trihydroxyl group on the B-ring and the gallate moiety have both been associated with increased free radical scavenging ability and, consequently, increased antioxidant activity (Taslimi et al., 2022; Wang et al., 2023). Earlier evidence suggests that modulation of oxidative stress by catechins (EGCG) can directly influence biofilm formation and microbial survival mechanisms (Zhang et al., 2023). This is generally attributed to H_2_O_2_ generation in addition to other mechanisms (Arakawa et al., 2004; Gopal et al., 2016).

**TABLE 8:** Antioxidant profile of catechins, gallocatchins and *Camellia sinensis*.

Sample	DPPH (IC_50_ µg/mL)	H_2_O_2_% inhibition	*Phospho-Molybdenum* (µg)	FRAP (µg)
Catechin	22.2 ± 0.04	68.2 ± 1.8	11.3 ± 0.04	18.2 ± 0.04
Epi-catechin gallate	8.5 ± 0.05	74.3 ± 1.4	4.5 ± 0.06	12.3 ± 0.06
Epi-catechin	12.4 ± 0.064	71.0 ± 1.4	10.2 ± 0.17	14.2 ± 0.03
Epi-gallocatechin	11.2 ± 0.02	76.2 ± 2.3	12.3 ± 0.03	25.3 ± 0.21
*Camellia sinensis*	64.2 ± 0.12	75.1 ± 2.6	32.5±	56.2 ± 0.31
Standard^1^	6.4 ± 0.02	77.4 ± 1.7	-	-

1 Ascorbic acid.

2 ascorbic acid equivalents.

#### Antimicrobial screening (MICs)

The minimum inhibitory concentration MIC of tested compounds was investigated by using the 96-well microplate method. It was evident that among all tested compounds, the epigallocatechin showed the highest inhibition against *Pseudomonas aeruginosa* (MIC 15.6 μg/mL), followed by *Klebsiella pneumoniae* (MIC 31.2 μg/mL), *Escherichia coli*, and *Staph aureus* (MIC 62.5 μg/mL) (Table 9). Only a moderate inhibition of catechin (MIC 62.5 μg/mL) against *S. aureus* and epi-catechin (MIC 62.5 μg/mL) against *K*. *pneumoniae* was recorded. All other compounds, including Camellia sinensis, were active at higher concentrations (MIC 15.6 μg/mL). Research has demonstrated that the trihydroxyphenyl group (gallo or gallate) significantly influences the regulatory activity of a catechin [40,41]. If the group is absent, as in (−)-epicatechin and (−)-catechin, activity is reduced or absent. However, when the group is present in the gallo form, where C1″ is directly linked to C2′, as in (−)-epigallocatechin and CG, it provides a regulating function (Wilhelm, 2008; VanˈT Slot and Humpf, 2009). Previous studies have demonstrated that epicatechin gallate induces reorganization of the cell membrane by effectively penetrating the hydrophobic core of bacterial lipid bilayers (Andrade et al., 2021; Sahadevan et al., 2023). Not only does this increase the net negative charge at the bacterial surface by reducing D-alanylation of the wall teichoic acid (Palacios et al., 2014; Taylor, 2020), but it also increases cell wall thickness.

**TABLE 9:** Antimicrobial activity (MIC µg/mL) of C. *sinensis* extract and components against bacterial strains.

Sample	*E.coli*	*K. pneumoniae*	*S. aureus*	*P*. *aeruginosa*
Catechin	>125 ± 0.0	>125 ± 0.0	62.5 ± 0.6	>125 ± 0.0
Epi-catechin gallate	62.5 ± 0.1	31.2 ± 0.2	62.5 ± 0.4	15.6 ± 0.0
Epi catechin	>125 ± 0.0	62.5 ± 0.6	>125 ± 0.0	>125 ± 0.0
Epi gallocatechin	>125 ± 0.0	>125 ± 0.0	>125 ± 0.0	>125 ± 0.0
C. *sinensis*	>125 ± 0.0	>125 ± 0.0	>125 ± 0.0	>125 ± 0.0
Standard*	24.4 ± 0.06	12.2 ± 0.2	48.8 ± 0.3	12.2 ± 0.07

Meropenem*.

Although epicatechin gallate reduced crystal-violet-stained biofilm biomass, this effect cannot be assigned solely to growth-independent anti-adhesion or biofilm-disruption activity. The crystal violet assay measures total attached biomass but does not distinguish reduced adhesion, biofilm viability, matrix disruption, or reduced bacterial growth (Kragh et al., 2019). Because epicatechin gallate also showed antibacterial activity and suppressed *P. aeruginosa* growth, the antibiofilm result is reported here as phenotypic biofilm inhibition. Future studies should include multi-concentration sub-MIC assays, growth normalization, biofilm viable-cell counts, metabolic assays, and microscopy to separate antibiofilm effects from antibacterial growth suppression (Martínez et al., 2019; Drumond et al., 2023).

#### Antibiofilm assay

**FIGURE 12:**
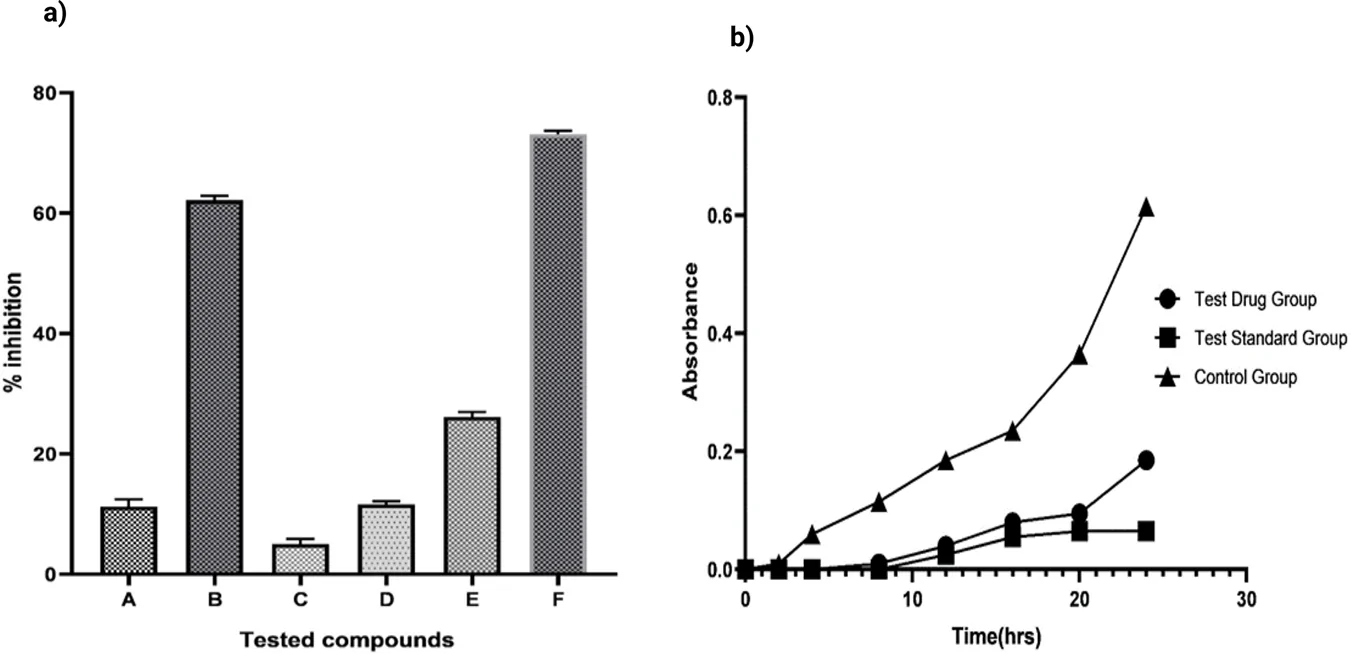
**(a)** Antibiofilm potential of catechin (A), epi-catechin gallate (B), epi-catechin(C), epi gallocatechin (D), *Camellia sinensis* (E), and standard drug (F). **(b)** Growth curve of *Pseudomonas aeruginosa* against epi-gallocatechin.

The antibiofilm assay used the biofilm-producing strain *P*. *aeruginosa* and for antibiofilm assay, Sub MIC values were used. It was evident that epi-catechin gallate showed a significant inhibition of biofilm formation (61.5% ± 2.3%), whereas very little or minor inhibition was recorded in the case of all other compounds (considered inactive). The C. *sinensis* extract, on the other hand, only presented a minor inhibition (25.4% ± 1.3%) (Figure 12). The C. *sinensis* catechins have long been investigated for their antibiofilm properties (Stapleton et al., 2004; Osterburg et al., 2009), and it has been reported earlier that epigallocatechin-3-gallate (EGCG), a major component of catechins (56%) in C. *sinensis,* had significant antibiofilm activities against *P*. *aeruginosa* (Palacios et al., 2014). Herein epicatechin gallate (ECG) and its analogues are reported to inhibit the biofilm formation and adhesion abilities of the bacterium decreased after treatment with ECG and its analogues in bacteria (Teng et al., 2025). However, the present antibiofilm assay did not directly measure LasR activity, autoinducer competition, quorum-sensing gene expression, or EPS/c-di-GMP pathway modulation; therefore, the observed biofilm inhibition can be interpreted as phenotypic antibiofilm activity rather than confirmed LasR-mediated competitive inhibition.

#### Growth curve

The inhibition level of *P*. *aeruginosa* was monitored for up to 24 h against epicatechin gallate. It was observed that up to the initial 8 h, the inhibition pattern of both epi-gallocatechin and ciprofloxacin was the same; however, afterward, comparably lower inhibition rates were recorded with epi-catechin gallate. Further, significant inhibition was observed for up to 20 h, followed by a gradual increase in bacterial growth (Figure 12). It was thus concluded that epicatechin gallate significantly inhibited bacterial growth for up to 20 h. A potent inhibition of bacterial growth with epicatechin gallate has been reported earlier, with up to 2 days of growth suppression observed, attributed to the suppression of DNA-binding protein function (Pavlik et al., 2023).

A key mechanistic limitation of this study is that LasR was assessed only by computational docking, whereas functional quorum-sensing activity was not directly measured. Therefore, the antibiofilm activity of epicatechin gallate should be interpreted as phenotypic biofilm inhibition, not as confirmed LasR inhibition or definitive quorum-sensing suppression. This distinction is important because validated quorum-sensing inhibition in *Pseudomonas aeruginosa* typically requires LasR/RhlR reporter assays, quorum-sensing-regulated virulence-factor measurements, biochemical receptor validation, or transcriptional analysis of quorum-sensing genes (Paczkowski et al., 2017; Ahmed et al., 2019). Moreover, biofilm reduction may occur through LasR-independent or indirectly LasR-associated mechanisms, including growth suppression, altered surface attachment, extracellular matrix modulation, cyclic-di-GMP-regulated adhesion, oxidative-stress responses, or broader Las/Rhl/Pqs network effects (Ha and O’Toole, 2015; Mukherjee et al., 2017). Future studies should include LasR-specific reporter assays, quantification of quorum-sensing-regulated virulence factors, autoinducer measurements, and expression analysis of *lasI*, *lasR*, *rhlI*, *rhlR*, and *pqs*-associated genes to determine whether epicatechin gallate directly modulates quorum sensing.

## Conclusion

This work presents an integrated phytochemical, computational and biological evaluation of catechins commonly reported in *C*. *sinensis*. Across assays, epicatechin gallate consistently emerged as the most promising lead, achieving the moderate biofilm inhibition against *P*. *aeruginosa*. The docking results can be interpreted as exploratory and do not establish competitive LasR binding. The observed antibiofilm activity may be associated with quorum-sensing-related processes or other mechanisms, including effects on bacterial growth, membrane-associated processes, oxidative stress responses, or extracellular matrix formation. Future studies should include native-ligand redocking, LasR reporter assays, quorum-sensing gene-expression profiling, EPS/c-di-GMP quantification, and testing against diverse clinical and polymicrobial biofilm models.

## Data Availability

The original contributions presented in the study are included in the article/supplementary material, further inquiries can be directed to the corresponding authors.
